# Supplementary material for: Drug repurposing for aging research using model organisms
Source: Aging Cell. 2017 Jun 16;16(5):1006–15. doi: 10.1111/acel.12626 (PMC5595691; doi:10.1111/acel.12626)
Supplement: Supplementary file 7 — Data S1 Zip‐Archive of all report cards. [file ACEL-16-1006-s007.zip › RC_XM1.pdf]

XM1

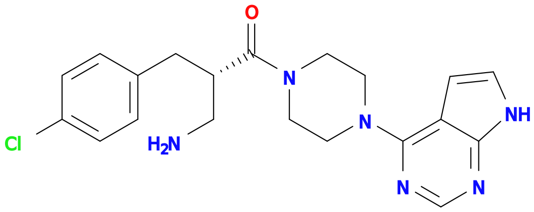

Database identifiers
ChEMBLCompound CHEMBL1236822

Ranking

	Rank	Score
Drosophila	246/697	0.653
C. elegans	98/591	0.324

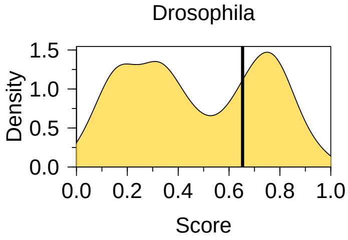

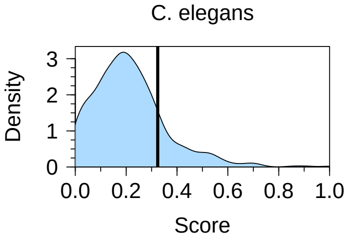

	Ageing implication	Domain conservation	Binding site conservation	Binding affinity	Bioavailability	Lipinski	Promiscuity	Purchasability	Drug approval	**Total**
Drosophila	1.0	0.869	0.899	0.93	(0.9)	0.0	-0.0	0.0	0.0	0.653
C. elegans	1.0	0.928	0.954	0.93	0.395	0.0	-0.0	0.0	0.0	0.324

Names

No synonyms found

Roles

ChEBI entry None has no roles

Status

Approved drug (according to ChEMBL)	No
Number of Rule of 5 violations	0
Binding affinity to original target in log units (RF-Score prediction)	7.58
Burns *C. elegans* bioavailability prediction	1.45

Compound Target Characteristics

RAC-alpha serine/threonine-protein kinase

Best gene implication in ageing for this target family came from gene Q94533 annotated in UniProt release 2014_02. Annotation GO 8340 (determination of adult lifespan) was Inferred from Mutant

Phenotype

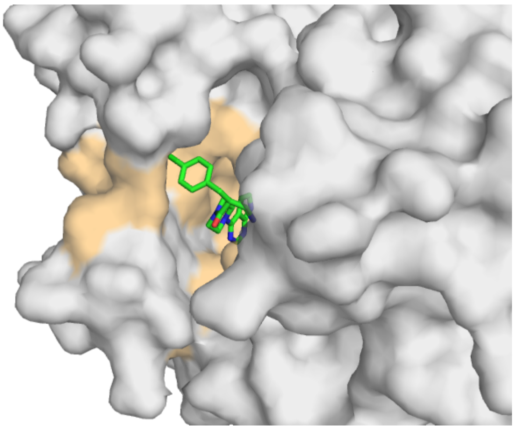

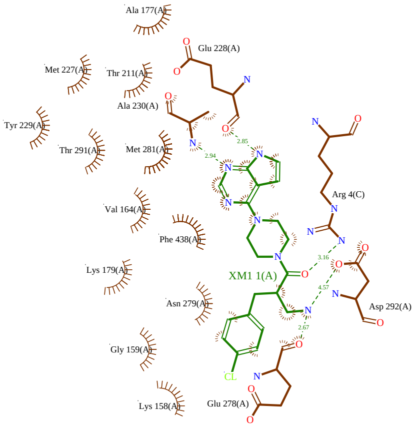

protein	amino acids contacts (binding site)																	
PDB:3ocb:chainA:P31749	K	G	G	K	V	A	K	T	M	E	Y	A	E	E	N	M	T	D	F
tr:B3KP61:B3KP61_HUMAN	K	G	G	K	V	A	K	T	M	E	Y	A	E	E	N	M	T	D	F
tr:B7Z5R1:B7Z5R1_HUMAN	K	G	G	K	V	A	K	T	M	E	Y	A	E	E	N	M	T	D	F
tr:B4DG79:B4DG79_HUMAN	K	G	G	K	V	A	K	T	M	E	Y	A	E	E	N	M	T	D	F
tr:M0R0P9:M0R0P9_HUMAN	K	G	G	K	V	A	K	T	M	E	Y	A	E	E	N	M	T	D	F
sp:P31749:AKT1_HUMAN	K	G	G	K	V	A	K	T	M	E	Y	A	E	E	N	M	T	D	F
tr:B0LPE5:B0LPE5_HUMAN	K	G	G	K	V	A	K	T	M	E	Y	A	E	E	N	M	T	D	F
sp:P31751:AKT2_HUMAN	K	G	G	K	V	A	K	T	M	E	Y	A	E	E	N	M	T	D	F
sp:P47196:AKT1_RAT	K	G	G	K	V	A	K	T	M	E	Y	A	E	E	N	M	T	D	F
tr:Q3HSE5:Q3HSE5_RAT	K	G	G	K	V	A	K	T	M	E	Y	A	E	E	N	M	T	D	F
tr:Q8CE74:Q8CE74_MOUSE	K	G	G	K	V	A	K	T	M	E	Y	A	E	E	N	M	T	D	F
sp:Q60823:AKT2_MOUSE	K	G	G	K	V	A	K	T	M	E	Y	A	E	E	N	M	T	D	F
tr:Q3TY95:Q3TY95_MOUSE	K	G	G	K	V	A	K	T	M	E	Y	A	E	E	N	M	T	D	F
sp:P31750:AKT1_MOUSE	K	G	G	K	V	A	K	T	M	E	Y	A	E	E	N	M	T	D	F
tr:H1ZYE3:H1ZYE3_DROME	K	G	G	K	V	A	K	V	L	E	Y	L	E	E	N	L	T	D	F
tr:Q94533:Q94533_DROME	K	G	G	K	V	A	K	V	L	E	Y	L	E	E	N	L	T	D	F
tr:P91656:P91656_DROME	K	G	G	K	V	A	K	V	L	E	Y	L	E	E	N	L	T	D	F
sp:Q9XTG7:AKT2_CAEEL	Q	G	G	K	V	A	K	T	M	E	F	A	E	E	N	L	T	D	F
sp:P11792:SCH9_YEAST	K	G	G	Q	V	A	K	V	T	D	Y	M	E	E	N	L	C	D	F

	whole protein		domain-based		contact-based	
protein	ident	simil	ident	simil	ident	simil
PDB:3ocb:chainA:P31749	0.99	1.0	0.99	1.0	1.0	1.0
tr:B3KP61:B3KP61_HUMAN	0.64	0.74	0.86	0.96	1.0	1.0
tr:B7Z5R1:B7Z5R1_HUMAN	0.87	0.87	1.0	1.0	1.0	1.0
tr:B4DG79:B4DG79_HUMAN	0.71	0.82	0.86	0.96	1.0	1.0
tr:M0R0P9:M0R0P9_HUMAN	0.65	0.76	0.79	0.9	1.0	1.0
sp:P31749:AKT1_HUMAN	1.0	1.0	1.0	1.0	1.0	1.0
tr:B0LPE5:B0LPE5_HUMAN	1.0	1.0	1.0	1.0	1.0	1.0
sp:P31751:AKT2_HUMAN	0.81	0.94	0.86	0.96	1.0	1.0
sp:P47196:AKT1_RAT	0.98	0.99	0.99	1.0	1.0	1.0
tr:Q3HSE5:Q3HSE5_RAT	0.81	0.94	0.86	0.96	1.0	1.0
tr:Q8CE74:Q8CE74_MOUSE	0.62	0.7	0.86	0.96	1.0	1.0
sp:Q60823:AKT2_MOUSE	0.81	0.94	0.86	0.96	1.0	1.0
tr:Q3TY95:Q3TY95_MOUSE	0.81	0.94	0.86	0.96	1.0	1.0
sp:P31750:AKT1_MOUSE	0.98	0.99	0.99	1.0	1.0	1.0
tr:H1ZYE3:H1ZYE3_DROME	0.3	0.57	0.42	0.78	0.79	0.9
tr:Q94533:Q94533_DROME	0.26	0.5	0.43	0.79	0.79	0.9
tr:P91656:P91656_DROME	0.21	0.4	0.43	0.79	0.79	0.9
sp:Q9XTG7:AKT2_CAEEL	0.48	0.75	0.59	0.86	0.84	0.95
sp:P11792:SCH9_YEAST	0.2	0.42	0.45	0.81	0.63	0.74

S6k (FBgn0015806) associated phenotypes

cell autonomous, chemical resistant, decreased cell number, decreased cell size, feeding behavior

defective, heat sensitive, increased cell growth, increased cell number, increased cell size, large body, long lived, memory defective, neuroanatomy defective, neurophysiology defective, nutrition conditional, partially, partially lethal - majority die, short lived, size defective, small body, somatic clone

(Information from FlyBase)

akt-2 (WBGene00000103) associated phenotypes

fat content increased, germ cell hypersensitive ionizing radiation, hypersensitivity to mutagen

(Information from WormBase)

akt-2 (UniProt:Q9XTG7) annotation

Function: Acts downstream of age-1 and pdk-1 in the daf-2/insulin receptor-like transduction pathway. Essential role in regulating developmental arrest at the dauer stage. Phosphorylates Forkhead-related daf-16 and the longevity-promoting skn-1 transcription factors, which inhibits their entry into the nucleus and antagonizes their functions. Role in immune function and pathogen resistance. (PubMed:10364160, PubMed:11381260, PubMed:11747825, PubMed:15068796, PubMed:18358814, PubMed:18782349, PubMed:9716402).

Cofactor:Mg(2+)

Enzyme regulation: Phosphorylated and activated by pdk-1. (PubMed:15068796).

Subunit: Interacts with pdk-1, sgk-1, akt-1 and daf-16. Part of a complex containing sgk-1, akt-1 and akt-2. (PubMed:15068796).

Tissue specificity: Expressed in neurons, muscle cells of the pharynx, rectal gland cells, and spermatheca. (PubMed:9716402).

Developmental stage: Expressed in late stage embryos and throughout life. (PubMed:9716402).

Disruption phenotype: Defective egg-laying and increased resistance to pathogens. Simultaneous knockdown of akt-1 and akt-2 result in dauer formation and a weak extension to life span. (PubMed:15068796, PubMed:18782349).

(Information from UniProt)
